# Supplementary figures and images for: Divergent thermal specialisation of two South African entomopathogenic nematodes
Source: PeerJ. 2015 Jul 2;3:e1023. doi: 10.7717/peerj.1023 (PMC4493674; doi:10.7717/peerj.1023)

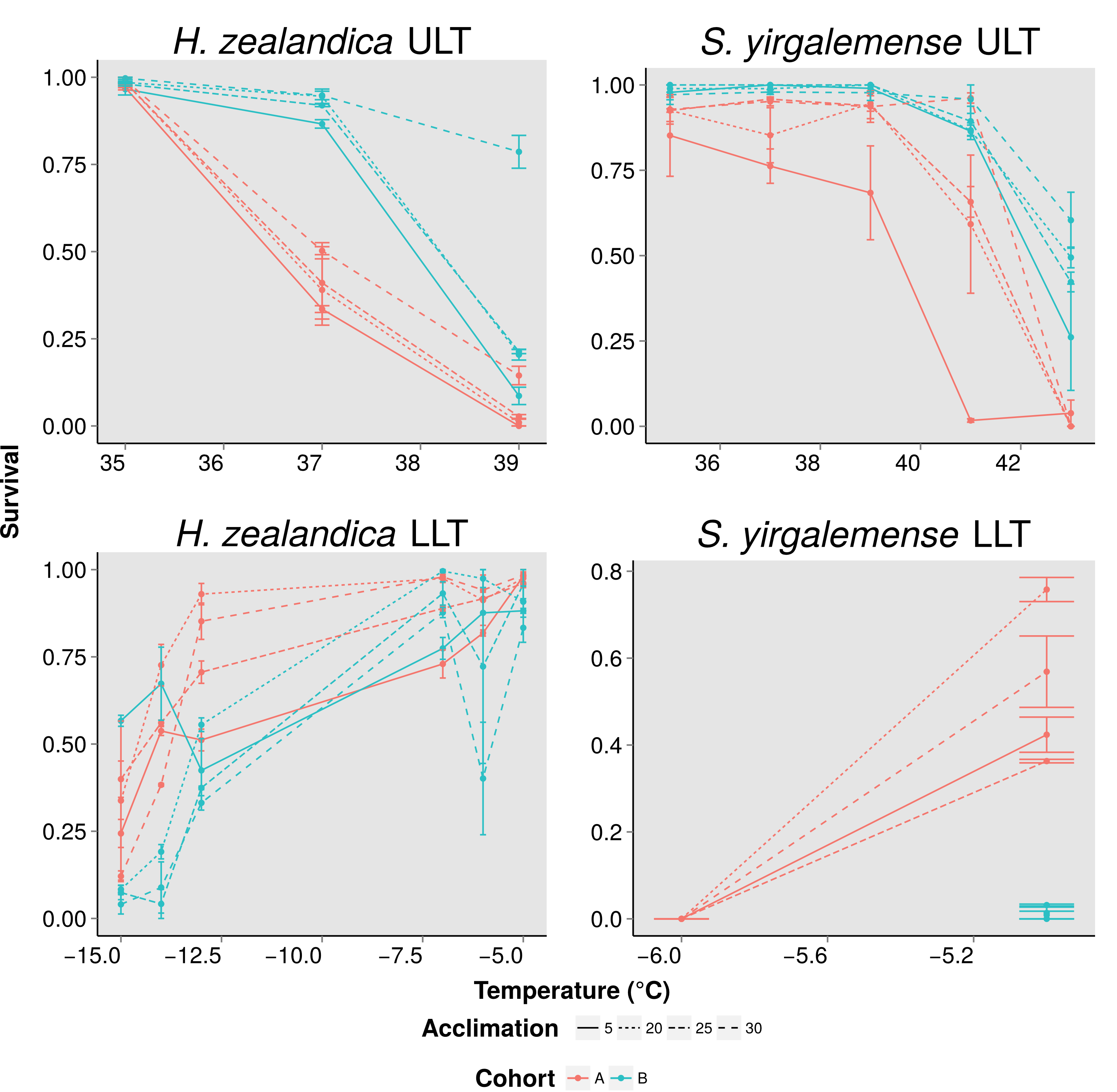

Supplement: Supplemental Information 1 — Error bars reflect ±1 standard error. Data represents the cohorts seperately: red indicates the first batch of experiments scored by MPH, blue indicates the second cohort of experiments scored by EL. [file peerj-03-1023-s001.png]
